# A detection panel of novel methylated DNA markers for malignant pleural effusion

**DOI:** 10.3389/fonc.2022.967079

**Published:** 2022-09-13

**Authors:** Chaonan Liang, Nan Liu, Qin Zhang, Mingming Deng, Jiangwei Ma, Jingwen Lu, Yan Yin, Jian Wang, Yuan Miao, Bin She, Qingchang Li, Gang Hou

**Affiliations:** ^1^Department of Cardio-Pulmonary Function, Henan Provincial People’s Hospital, Zhengzhou University People’s Hospital, Henan University People’s Hospital, Zhengzhou, Henan, China; ^2^Department of Pulmonary and Critical Care Medicine, The First Hospital of China Medical University, Shenyang, China; ^3^Department of Pathology, The First Hospital and College of Basic Medical Sciences, China Medical University, Shenyang, China; ^4^Department of Pulmonary and Critical Care Medicine, China-Japan Friendship Hospital, National Center of Respiratory Medicine, Institute of Respiratory Medicine, Chinese Academy of Medical Sciences, National Clinical Research Center for Respiratory Diseases, Beijing, China; ^5^Academic Development, Tellgen Corporation, Shanghai, China

**Keywords:** DNA methylation, pleural effusion, biomarkers, *SHOX2*, *RASSF1A*, *SEPTIN9*, *HOXA9*

## Abstract

**Background:**

Cytology remains the gold standard for the detection of malignant cells in pleural effusion. However, its sensitivity is limited. The aim of this study was to establish a novel panel of cancer-specific methylated genes for the differential diagnosis of malignant pleural effusion (MPE).

**Methods:**

A cohort of 100 cancer patients (68 lung cancer, 32 other malignant tumors) and 48 patients with benign disease presenting with pleural effusion was prospectively enrolled. Pleural effusion was evaluated by means of cytopathological investigation and DNA methylation of SHOX2, RASSF1A, SEPTIN9 and HOXA9 in the cellular fraction. DNA methylation in bisulfite-converted DNA was determined using quantitative methylation-specific real-time PCR (MS-PCR). Cytopathological and DNA methylation results were evaluated with regard to the final clinical diagnosis.

**Results:**

The LungMe^®^ SHOX2 and RASSF1A Assay (Tellgen Corporation, China) has been reported to be highly sensitive and specific for lung cancer using bronchial aspirates. As expected, LungMe^®^ detected metastases of lung cancer (sensitivity: 76.5%) as well as metastases of other malignant tumors (sensitivity: 68.8%). OncoMe, a novel combination of SHOX2, RASSF1A, SEPTIN9 and HOXA9 methylation, led to an additional 11% increase in the detection rate of MPE, resulting in a sensitivity of 85% and a specificity of 96%. Overall, OncoMe showed a higher positive detection rate in SCLC (100%), LUAC (87%), OC (100%), BC (92.9%), GC (80.0%), and MESO (80%) than in LUSC (50%). Cytopathological analyses only detected 23 positive samples, which were all positively measured by both LungMe^®^ and OncoMe.

**Conclusion:**

OncoMe has potential for use as a biomarker for the detection of MPE, even not limited to lung cancer.

## Introduction

Although epidemiological studies in China are not available, the annual incidence of pleural effusion in the United States of America is estimated to be more than 1,500,000 (1). Lung cancer is the most common cause of malignant pleural effusion (MPE), accounting for approximately 1/3 of MPE cases, followed by breast cancer, ovarian cancer and gastrointestinal cancer; the primary tumor cannot be found in 5%-10% of MPE cases (2, 3). Nonmalignant etiologies of pleural effusion include congestive heart failure, tuberculous pleuritis, pneumonia, pulmonary embolism or infarction, cirrhosis and collagen disease (3). Pleural effusion has a wide differential diagnosis. A delayed etiological diagnosis will directly affect the subsequent treatment of patients and can be associated with markedly higher morbidity and mortality.

In routine clinical practice, investigation of the cause of pleural effusion begins with obtaining the patients’ clinical history followed by performing a physical examination. The accurate and early detection of tumor cells in the pleural effusion is of strong clinical importance in the differential diagnosis of MPE. In approximately 50% of lung cancers (4) and 60% of all cancers (5), the malignant nature of a pleural effusion can be confirmed cytologically. The yield of positive tumor diagnoses is highest for adenocarcinoma and lower for mesothelioma, squamous cell carcinoma, lymphoma, and sarcoma (3). One study showed that cytopathological investigation with a second specimen can yield an additional 27% increase in the positive rate (4). However, a gray zone always exists, where the cytopathologist encounters problems in determining the nature of the cells, whether reactive, atypical, or beyond doubt malignant. Thus, there is a need for additional methods, preferentially on the same pleura fluid, to prevent repeated diagnostic efforts and harm to patients.

A number of studies have demonstrated frequent promoter methylation in lung cancer cells, including short stature homeobox gene two (*SHOX2*), RAS association domain family 1, isoform A (*RASSF1A*) (6–8) and homeobox A9 (*HOXA9*) (9, 10). *HOXA9* promoter hypermethylation has also been observed in a large proportion of high-grade serous ovarian carcinomas (11). Quantitative *SHOX2* and Septin 9 (*SEPTIN9)* methylation levels have been successfully applied for the diagnosis of colonic adenomas (12) and the detection of malignant cells in pleural effusions (13) and ascites (14). We previously reported that the combination of *SHOX2* and *RASSF1A* methylation in bronchoalveolar lavage fluid (BALF) yielded a diagnostic sensitivity of 71.5-81.0% and a specificity of 90-97.4% (6, 7). This led to the development of the LungMe^®^ Assay (Tellgen Corporation, Shanghai, China), an *in vitro* diagnostic test kit to aid pathologists in the diagnosis of lung cancer.

The detection of promotor hypermethylation of tumor suppressor genes in the pleural effusion cellular fraction might aid greatly in the differential diagnosis of MPE. We therefore determined the promoter methylation status of the four genes in 148 patients with pleural effusions caused by various diseases, including lung adenocarcinoma (LUAC), lung squamous carcinoma (LUSC), breast cancer (BC), ovarian carcinoma (OC), gastrointestinal cancer (GC), malignant mesothelioma (MESO), pneumonia (PNE), and tuberculous pleuritis (TB), to examine whether this panel of novel methylated DNA markers could effectively identify malignant effusions.

## Methods

### Ethics, consent, and permissions

This study was approved by the institutional ethical review board of the First Hospital of China Medical University (Reference number: AF-SOP-07-1.1-01) and was supported by the Nonprofit Central Research Institute Fund of Chinese Academy of Medical Sciences (2020-PT320-001).

### Patients

Pleural effusion samples from patients under investigation for suspected cancer at the First Hospital of China Medical University (Shenyang, China) between 10/2019 and 05/2021 were included in this study. Conventional cytopathological investigation and the DNA methylation of *SHOX2*, *RASSF1A*, *SEPTIN9* and *HOXA9* were measured in the cellular fraction of pleural effusion samples from 148 patients in this cohort study. Patient characteristics are summarized in **Table 1**. Among them, 100 cases were diagnosed as cancer, including 68 lung cancer, 14 BC, 5 OC, 5 GC, 5 MESO and three other malignant carcinoma cases. The other 48 cases were exclusively nonmalignant benign diseases, including 23 pneumonia, 22 tuberculous and three cirrhosis cases. Detection of malignancy was performed by histological analysis based on biopsy or surgical specimens

**Table 1:** Baseline characteristics of patients.

	Malignant PE							Benign PE			
	LC (n = 68)	BC (n = 14)	OC (n = 5)	GC (n = 5)	MESO (n = 5)	Others MPE (n = 3)	Total MPE (n = 100)	PNE (n = 23)	TB (n = 22)	Cirrhosis (n = 3)	Total BPE (n = 48)
Age (years)											
Mean ± SEM	61.6 ± 13.4	53.6 ± 20.2	55.4 ± 11.5	57.2 ± 13.2	68.4 ± 6.2	46.7 ± 19.1	59.6 ± 13.9	59.4 ± 11.3	51.5 ± 16.4	56.8 ± 13.9	56.1 ± 14.5
Range	25-92	32-76	46-75	43-73	58-73	25-61	25-92	25-79	24-91	38-77	24-91
Gender											
Female (%)	29 (42.6)	14 (100)	5 (100)	2 (40)	2 (40)	2 (66.7)	55 (55)	6 (26.1)	6 (27.3)	2 (66.7)	14 (29.2)
Male (%)	39 (57.4)	0 (0)	0 (0)	3 (60)	3 (60)	1 (33.3)	46 (46)	17 (73.9)	16 (72.7)	1 (33.3)	34 (70.8)

PE, Pleural effusion; LC, Lung Cancer; BC, Breast Cancer; OC,Ovarian Cancer; GC, Gastrointestinal Cancer; MESO, Mesothelioma; Others MPE (n = 3); Metastasis Tumor (n = 1); Hematonosis (n = 2); PNE, Pneumonia; TB, Tuberculosis.

### Pleural effusion sample collection and processing

Fresh pleural effusion (5-20 ml) samples were fixed with 20 ml of cell prevention solution (20140074, Tellgen Co., China) and centrifuged at 4,000 x g for min at 24°C. The pellets were dissolved in 1 ml of cell prevention solution and stored at room temperature for no more than 2 weeks.

### DNA extraction, bisulfite treatment and methylation analysis

DNA extraction and DNA bisulfite conversions of the cell pellets were performed using the Methy-All-In-One Kit (Tellgen Co., Shanghai, China) as described earlier (9, 11, 15). The concentration of extracted DNA was accurately measured using highly sensitive fluorescent dye assays (Fluo-100B, Hangzhou Allsheng Instruments Co., Ltd., China). Fifty nanograms of DNA/sample was treated with sodium bisulfite using the Tellgen DNA Purification Kit (PF03X056, Tellgen Co., China). After purification, the bisulfite-converted DNA was parallel amplified in two tubes by multiple methylation-specific real-time PCR (MS-PCR). One MS-PCR amplifies methylated *SHOX2*(VIC), *RASSF1A*(FAM), and *-ACTB*(CY5) DNA, while another MS-PCR amplifies methylated *HOXA9*(VIC), *SEPTIN9*(FAM), and *-ACTB*(CY5), which served as internal controls for the quantification of total input DNA.

Primer and probe sequences were as follow, the forward primer of SHOX2 was TTGTTTTTGGGTTCGGGTT, the reverse primer of SHOX2 was CATAACGTAAACGCCTATACTCG, the probe of SHOX2 was VIC-ATCGAACAAACGAAACGAAAATTACC, the forward primer of RASSF1A was CGGGGTTCGTTTTGTGGTTTC, the reverse primer of RASSF1A was CCGATTAAATCCGTACTTCGC, the probe of RASSF1A was FAM-TCGCGTTTGTTAGCGTTTAAAGT, the forward primer of HOXA9 was CGTTTAGGGAGTATCGCGGGTGTAG, the reverse primer of HOXA9 was CGTTTAGGGAGTATCGCGGGTGTAG, the probe of HOXA9 was VIC-CCCTCCTAACCAACTCCTCCGTAA, the forward primer of SEPTIN9 was GTTTTGTATTGTAGGAGCGC, the reverse primer of SEPTIN9 was CGAAAAAACGCCCCCGACGA, the probe of SEPTIN9 was FAM-AACCCTACGCGCTAA.

The positive quality controls were plasmids containing the methylated DNA of *SHOX2*, *RASSF1A*, *SEPTIN9* and *HOXA9* that have no bioactivity. PCR amplification was performed in an ABI 7500 Real-Time PCR instrument (Applied Biosystems, CA, USA), and SDS Software (Applied Biosystems) was used to obtain the results of the analysis. Co-methylation levels of a gene of interest were expressed by ΔCt, where ΔCt = Ct (gene of interest) - Ct (internal control). Samples were included in the analysis when 18 ≤Ct*_-ACTB_* ≤30. Samples were classified as methylation positive when at least one of the four genes’ DNA methylation levels correspondingly met the following quantitative criteria: Ct*_SHOX2_* <32 and ΔCt*_SHOX2_* ≤ 9; Ct*_RASSF1A_* <35 and ΔCt*_RASSF1A_* ≤ 12; Ct*_SEPTIN9_* <35 and ΔCt*_SEPTIN9_ ≤*9; and Ct*_HOXA9_* <32 and ΔCt*_HOXA9_ ≤*8. All others were classified as methylation negative.

### Statistical analysis

Statistical analyses were performed using the SPSS 19.0 software package (SPSS Inc., Chicago, IL). The frequency of methylation in the *SHOX2*, *RASSF1A*, *SEPTIN9* and *HOXA9* genes was analyzed using the chi-square test. For each diagnostic marker, we established a receiver operating characteristic (ROC) curve to calculate the area under the ROC curve (AUC) to evaluate the diagnostic efficacy. A P value <0.05 was considered statistically significant.

## Results

### Methylation frequency and association with clinicopathologic features in pleural effusion samples

**Figure 1:**
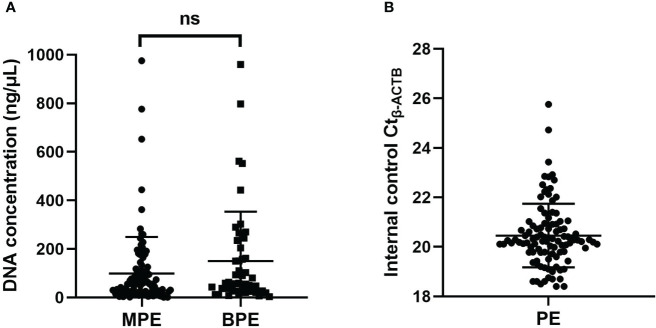
**(A)** The concentration distribution of pleural effusion between MPE and BPE. **(B)** The distribution of the Ct value of the internal control *b-ACTB.* ns, not significant; MPE, malignant pleural effusion; BPE, benign pleural effusion; PE, pleural effusion.

To confirm the cancer specificity of the promoter methylation events of these four genes, an MS-qPCR assay for each of the genes was conducted on 100 malignant pleural effusion (MPE) and 48 nonmalignant benign pleural effusion (BPE) samples. The DNA concentration of pleural effusion was distributed in a very wide range from 1 ng/µL to 960 ng/µL. The frequency distribution of pleural fluid concentration is illustrated in **Supplementary Figure 1**. The amplification curve of *SHOX2*, *RASSF1A*, *SEPTIN9* and *HOXA9* promoter methylation was shown in **Supplementary Figure 2**. There was no significant difference in the concentration distribution between MPE and BPE (**Figure 1A**). Through accurate concentration determination, the same amount of DNA (50 ng) was added to each PCR unless the total amount of DNA was insufficient. The detection of the internal control ACTB was very stable, and its measurement value fluctuated in a very small range, with Ct*_-ACTB_* = 20.48 ± 1.28 (**Figure 1B**). A valid measurement (18 ≤Ct*_-ACTB_ ≤*30) was achieved for all 148 specimens.

**Figure 2:**
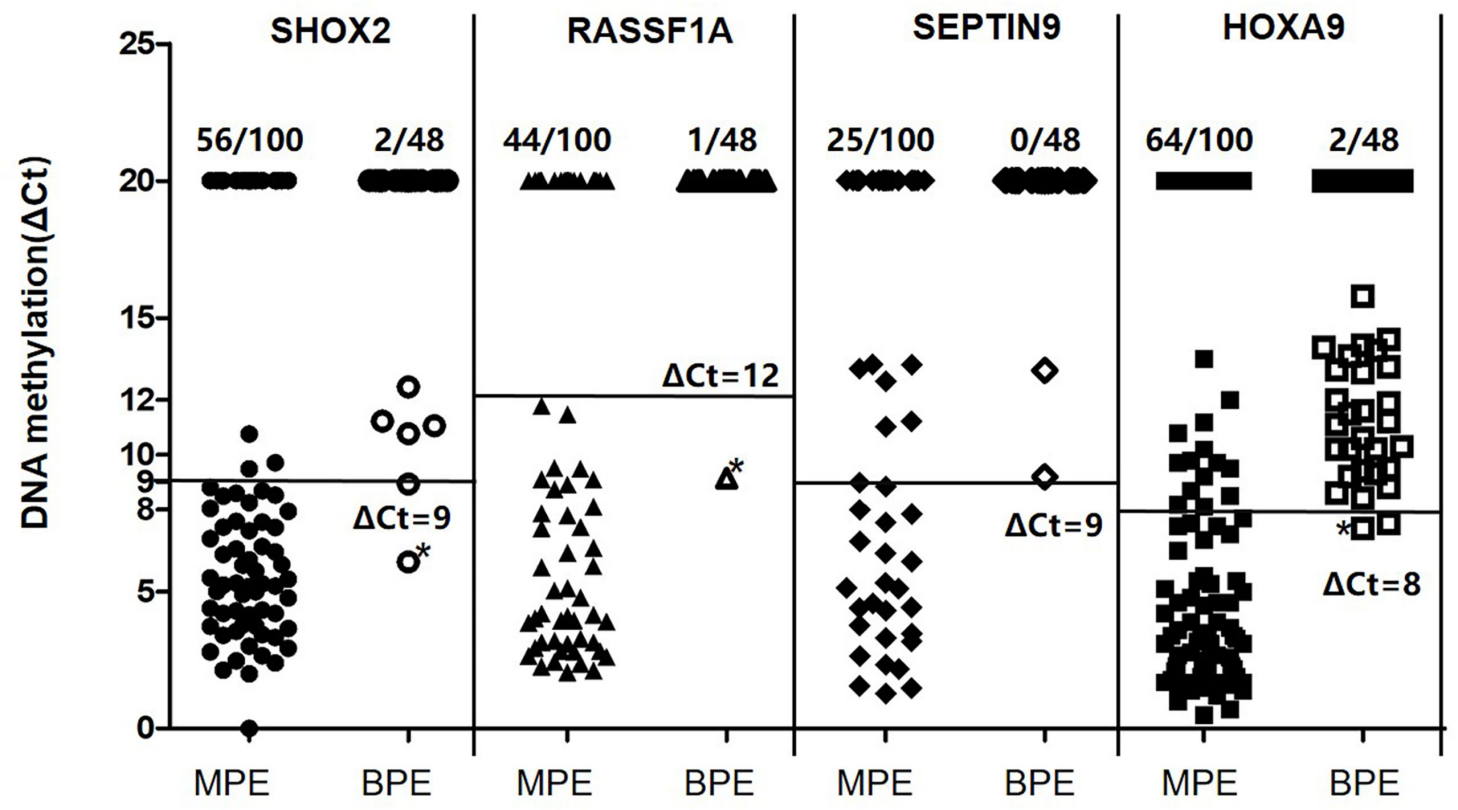
Quantitative analysis of SHOX2, RASSF1A, SEPTIN9 and HOXA9 DNA methylation in MPE (n = 100) and BPE (n = 48) specimens. MPE, malignant pleural effusion; BPE, benign pleural effusion; SHOX2, short stature homeobox gene two; RASSF1A, RAS association domain family 1, isoform A; HOXA9, homeobox A9; SEPTIN9, Septin 9.

**Figure 3:**
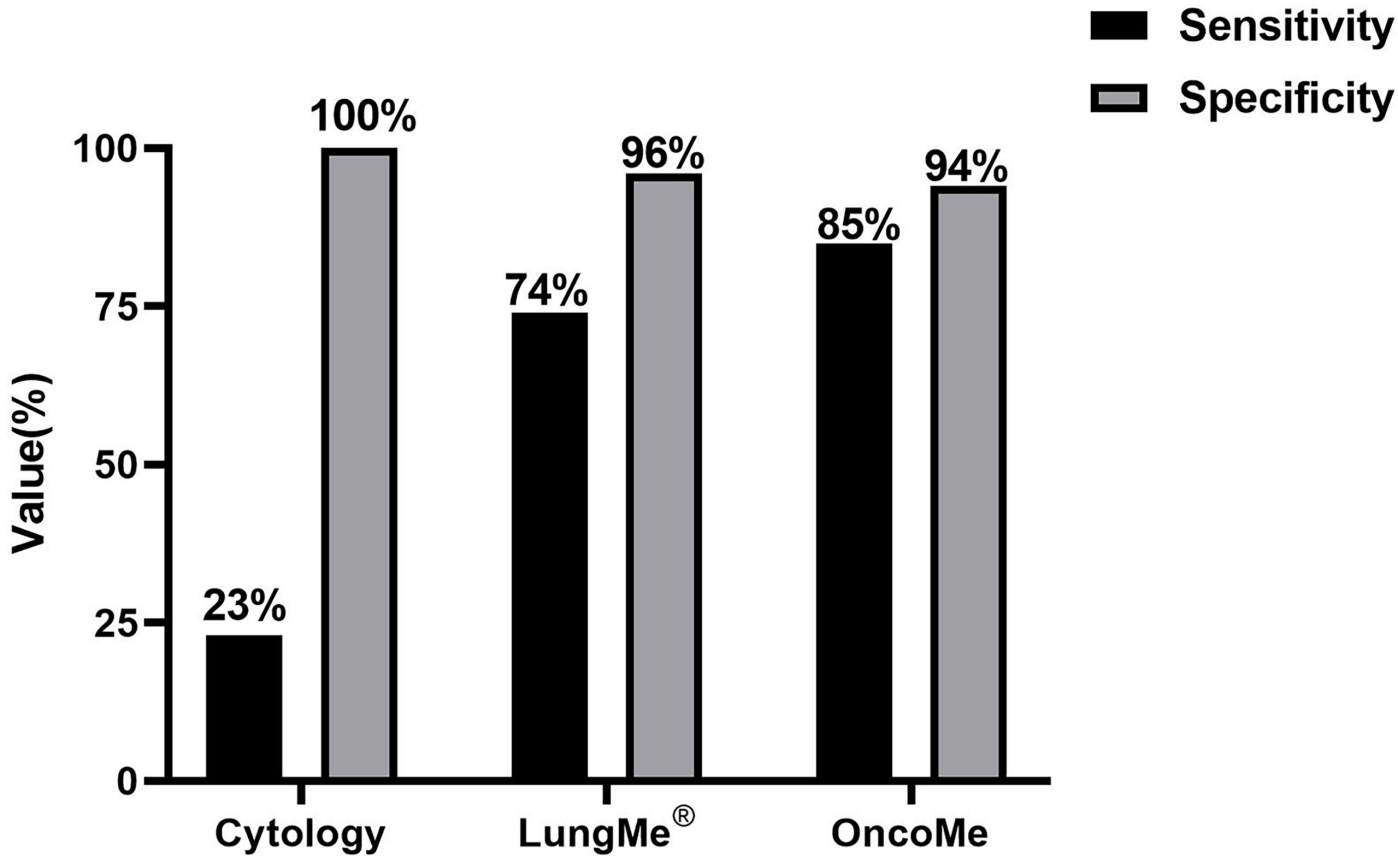
Comparison of cytology and the gene methylation panels LungMe^®^ (SHOX2+RASSF1A) and OncoMe (SHOX2+RASSF1A+SEPTIN9+HOXA9) for the differential diagnosis of pleural effusion.

By using the optimal methylation cutoff value for individual genes, the observed sensitivity and specificity of an individual gene for MPE detection ranged from 25% to 64% and from 95.8% to 100%, respectively (**Figure 2**). The cytological examination of these specimens achieved 23% sensitivity and 100% specificity. Combined detection of SHOX2 and RASSF1A (LungMe^®^) led to 74% sensitivity and 96% specificity. By adding SEPTIN9 and HOXA9, the detection rate of OncoMe increased to 85%, while the specificity slightly decreased to 94% (**Figure 3**).

For all samples, the clinical performance of the methylation events of these four genes with regard to pathologically determined histological classification was analyzed and is detailed in **Table 2**. The positive detection rates of each marker in lung carcinomas ranked from high to low were 69.1% (HOXA9), 64.7% (SHOX2), 45.6% (RASSF1A), 30.9% (cytology), and 20.6% (SEPTIN9). Furthermore, LungMe^®^ alone allows for the detection of small cell lung carcinoma (SCLC) with a high sensitivity of 100%. Combined with HOXA9 and SEPTIN9, the detection rates in lung adenocarcinoma (LUAC) were greatly improved from 79.7% to 87.0%. However, a lower sensitivity of LungMe^®^ was observed with lung squamous carcinoma (LUSC), which was improved by adding SEPTIN9 (increased from 33.3% to 50.0%). Interestingly, the detection rate of SEPTIN9 in SCLC was 0%, while the other three genes and cytology all showed the highest sensitivity for SCLC among different subgroups of MPEs, ranging from 75% to 100%.

**Table 2:** Diagnostic yield of cytology and methylation analyses in different subgroups of MPEs.

Tumor classification		Cytolog+n (%)	SHOX2+n (%)	RASSF1A+n (%)	SEPTIN9+n (%)	HOXA9+n (%)	LungMe^®^+n (%)	OncoMe+n (%)
Lung cancer								
LUAC	(n = 54)	17 (25.9)	35 (64.8)	25 (46.3)	11 (20.4)	40 (74.1)	43 (79.7)	47 (87.0)
LUSC	(n = 6)	0 (0.0)	2 (33.3)	0 (0.0)	2 (33.3)	1 (16.7)	2 (33.3)	3 (50.0)
SCLC	(n = 4)	3 (75.0)	4 (100)	4 (100)	0 (0.0)	3 (75.0)	4 (100)	4 (100)
undefined LUC	(n = 4)	0 (0.0)	3 (75.0)	2 (50.0)	1 (25.0)	3 (75.0)	3 (75.0)	3 (75.0)
Total	(n = 68)	21 (30.9)	44 (64.7)	31 (45.6)	14 (20.6)	47 (69.1)	52 (76.5)	57 (83.9)
Non-lung cancer								
BC	(n = 14)	0 (0.0)	5 (35.7)	8 (57.1)	8 (57.1)	8 (57.1)	10 (71.5)	13 (92.9)
OC	(n = 5)	0 (0.0)	1 (20.0)	3 (60.0)	0 (0.0)	3 (60.0)	4 (80.0)	5 (100)
GC	(n = 5)	1 (20.0)	2 (40.0)	0 (0.0)	3 (60.0)	3 (60.0)	2 (40.0)	4 (80.0)
MESO	(n = 5)	1 (20.0)	3 (60.0)	1 (20.0)	0 (0.0)	3 (60.0)	4 (80.0)	4 (80.0)
Others MPE	(n = 3)	0 (0.0)	1 (33.3)	1 (33.3)	0 (0.0)	0 (0.0)	2 (66.7)	2 (66.7)
Total	(n = 32)	2 (6.3)	12 (37.5)	13 (40.6)	11 (34.4)	17 (53.1)	22 (68.8)	28 (87.5)
Total (MPEs)	(n = 14)	23 (23)	56 (56)	44 (44)	25 (25)	64 (64)	74 (74)	85 (85)

**LungMe^®^ +:** SHOX2 positive or RASSF1A positive**; OncoMe+:** SHOX2 positive or RASSF1A positive or SEPTIN9 positive or HOXA9 positive**; LUAC**: Lung adenocarcinoma**; LUSC:** Lung squamous carcinoma; **SCLC:** Small Cell Lung Carcinoma; **BC**: Breast Cancer; **OC**: Ovarian Cancer; **GC**: Gastrointestinal Cancer; **MESO**: Mesothelioma; **Others MPE** (n=3); Metastasis Tumor (n = 1); Hematonosis (n = 2).

Although the detection rates of individual genes were mostly significantly different between lung cancer and malignant tumors of other origins, the difference was eliminated by the combination of different genes, such as LungMe^®^ (p= 0.467) or OncoMe (p=0.769).

The positive detection rates of LungMe^®^ and OncoMe in BC were 71.5% and 92.9%, respectively, despite the observed sensitivity of an individual gene ranging between 35.7% (SHOX2) and 57.1% (RASSF1A, SEPTIN9, HOXA9). The high sensitivity of LungMe^®^ was also observed with OC, which was improved by adding HOXA9 (increased from 83.3% to 100%). By adding SEPTIN9, the detection rate of LungMe^®^ in GC was improved from 40% to 80%. The detection rates of LungMe^®^ in MESO and other MPEs were 80% and 66.7%, respectively, which could not be further improved by combining with either SEPTIN9 or HOXA9. No promotor methylation of SEPTIN9 was detected in OC or MESO.

### Comparison of SHOX2, RASSF1A, SEPTIN9 and HOXA9 methylation levels in individual MPE specimens

**Figure 4:**
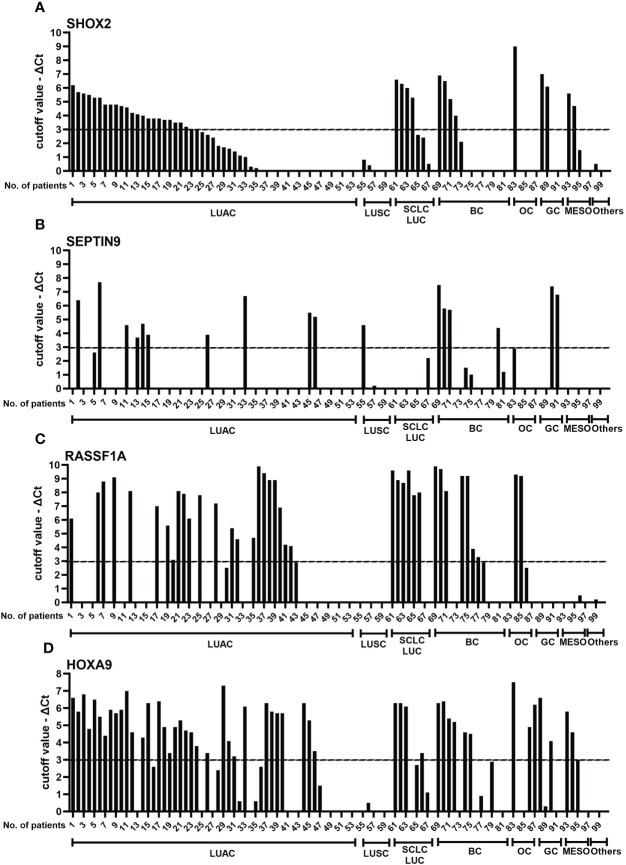
Comparison of SHOX2 **(A)**, SEPTIN9 **(B)**, RASSF1A **(C)**, and HOXA9 **(D)** methylation levels in individual pleural effusion specimens. To display the change in methylation levels more intuitively, the methylation level of an individual gene was calculated as the X value, which was calculated as 9-ΔCtSHOX2, 12-ΔCtRASSF1A, 8-ΔCtHOXA9 and 9-ΔCtSEPT9. Then, the methylation levels of SHOX2, RASSF1A, HOXA9 and SEPTIN9 were categorized as strongly methylated (X>3), mildly methylated (0<X ≤ 3) and nonmethylated (X = 0). LUAC, lung adenocarcinoma; LUSC, lung squamous carcinoma; SCLC, small cell lung carcinoma; BC, breast cancer; OC, ovarian carcinoma; GC, gastrointestinal cancer; MESO, malignant mesothelioma; SHOX2, short stature homeobox gene two; RASSF1A, RAS association domain family 1, isoform A; HOXA9, homeobox A9; SEPTIN9, Septin 9.

Next, to assess how individual genes complement each other to improve diagnostic accuracy in different subgroups of MPE, a comparison of *SHOX2*, *RASSF1A*, *SEPTIN9* and *HOXA9* promoter methylation levels in individual pleural effusion specimens was performed, as shown in **Figure 4**. To display the change in methylation level more intuitively, the methylation level of an individual gene was displayed as the X value, which was calculated as “cutoff value - ΔCt”, namely, 9-ΔCt_SHOX2_, 12-ΔCt_RASSF1A_, 8-ΔCt_HOXA9_ and 9-ΔCt_SEPTIN9_ for the individual genes. Furthermore, the association of semiquantitative individual gene methylation status was analyzed and is plotted in **Figure 4**. The methylation level of an individual gene was categorized as strongly methylated (X>3), mildly methylated (0<X ≤ 3), or nonmethylated (X=0). The semiquantitative data revealed that these four genes each have their own expression profiles, but they also more or less interrelate with each other.

The performance of *SHOX2* and *HOXA9* gene promoter methylation showed the best consistency (77%), followed by *HOXA9/RASSF1A* (71%, see **Table 1**). However, the combination panels of S*HOX2/RASSF1A* and *HOXA9/RASSF1A* both detected the most subjects with tumors (74/100, 74%). The *HOXA9* gene complements the LungMe^®^ combination mainly in LUAC, BC and OC, while by adding *SEPTIN9*, the detection rate of LungMe^®^ increased in LUAC, LUSC, BC and GC. No promotor methylation was detected in LUSC with *RASSF1A* or in OC or MESO with *SEPTIN9* (**Figure 4**). Overall, the promoter methylation analysis of *SHOX2* in MPE showed a medium detection sensitivity of 56/100. Combined with *RASSF1A*, the detection rate increased to 74/100, which was further improved by adding *HOXA9* (81/100). Finally, for OncoMe, the methylation pattern spectrum of these four genes led to a sensitivity of 85% (85/100).

### ROC curve analysis of the LungMe^®^ and OncoMe methylation panels in PE

**Figure 5:**
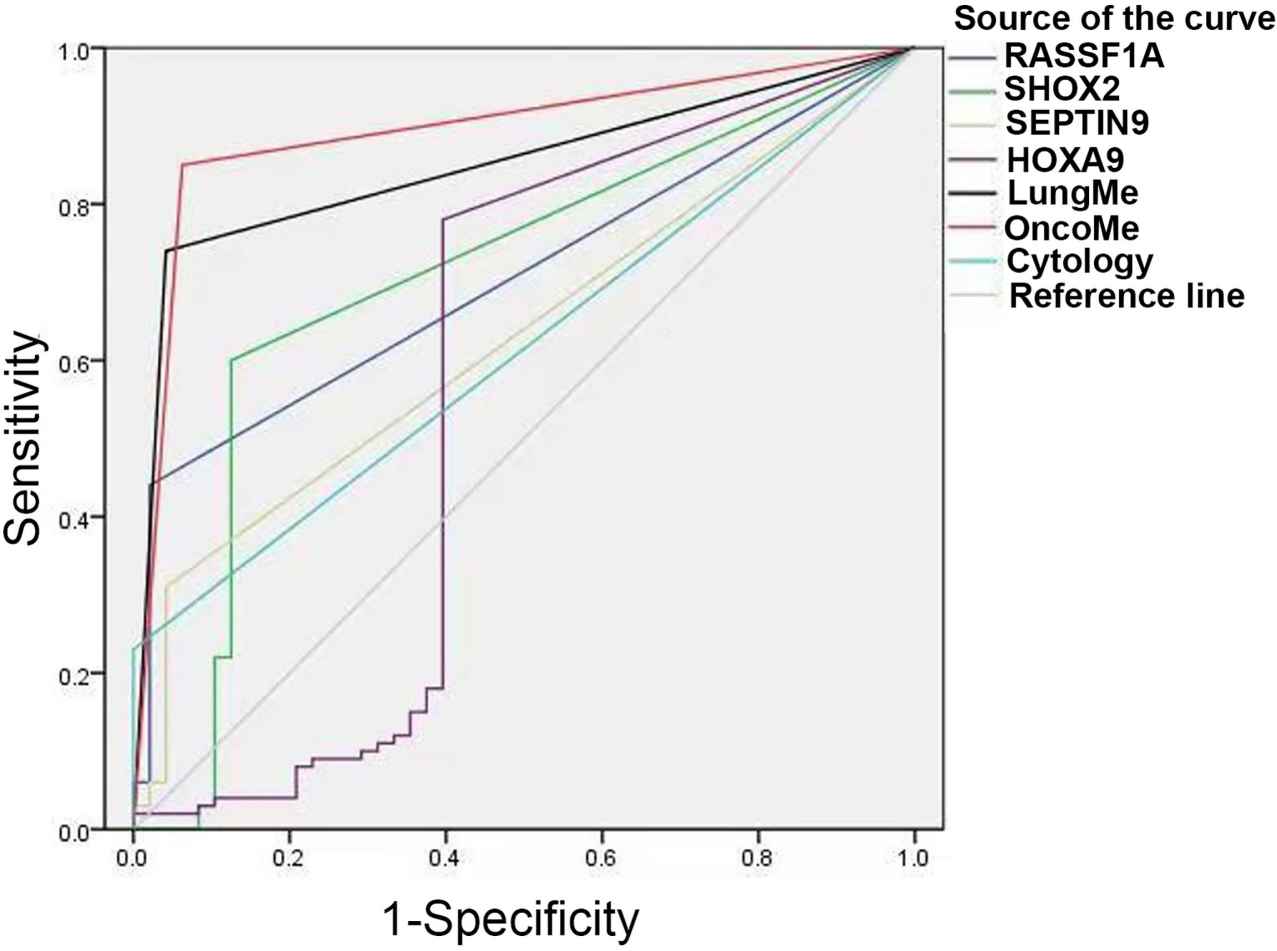
ROC curves for cytology, SHOX2, RASSF1A, SEPTIN9, HOXA9 and the LungMe^®^ and OncoMe methylation panels. SHOX2, short stature homeobox gene two; RASSF1A, RAS association domain family 1, isoform A; HOXA9, homeobox A9; SEPTIN9, Septin 9.

ROC curve analysis was performed to compare the diagnostic efficacy of the four individual genes and two methylation panels in PE. As shown in **Figure 5** and **Table 3**, the LungMe^®^ and OncoMe methylation panels in pleural effusion showed the highest AUC values of 0.849 and 0.894, respectively, compared to cytology (AUC value: 0.615) and individual methylation markers. In addition, OncoMe showed the highest diagnostic sensitivity of 85%, followed by LungMe^®^ (74%), compared to cytology (23%), *SHOX2* (56%), *RASSF1A* (44%), *SEPTIN9* (25%), and *HOXA9* (64%). All the methylation markers, including the two panels, showed a very high positive predictive value, ranging from 96.6% to 100%, which suggested that *SHOX2*, *RASSF1A*, *SEPTIN9*, and *HOXA9* promoter methylation detection in pleural effusion could be effective complementary tools for cytology in the differential diagnosis of MPE.

**Table 3:** The diagnostic efficacy of cytology, SHOX2, RASSF1A, SEPTIN9, HOXA9 and LungMe^®^、OncoMe methylation panel.

	AUC		Cut-off Value	Sensitivity	Specificity	PPV	NPV
	Value	95%CI					
**Cytology**	0.615	0.525-0.705	/	23.0%	100.0%	100.0%	38.4%
**SHOX2**	0.705	0.611-0.800	ΔCt ≤ 9	56.0%	95.8%	96.6%	51.1%
**RASSF1A**	0.706	0.624-0.789	ΔCt ≤ 12	44.0%	97.9%	97.8%	45.6%
**SEPTIN9**	0.630	0.539-0.720	ΔCt ≤ 9	25.0%	100%	100%	39.0%
**HOXA9**	0.565	0.445-0.685	ΔCt ≤ 8	64.0%	95.8%	97.0%	56.1%
**LungMe**	0.849	0.786-0.913	/	74.0%	95.8%	97.4%	63.9%
**OncoMe**	0.894	0.836-0.951	/	85.0%	93.8%	96.6%	75.0%

Various age-related DNA methylation changes have been described, such as differential and variable methylation. Therefore, we analyzed the diagnostic value of LungMe and OncoMe in patients of different ages. We divide patients into young individuals (between 25 and 50 years old, n= 27) seems higher than in older patients (≥ 50 years old, n= 121). As shown in **Supplementary Figure 3**, the AUC value of LungMe^®^ methylation panel in older patients were 0.823 (p<0.001), and in young individuals was 0.971 (p<0.001). The AUC value of OncoMe methylation panel in older patients was 0.877 (p<0.001), and in young individuals was 0.971 (p<0.001).

## Discussion

The performance of the LungMe^®^ (*SHOX2* and *RASSF1A*) methylation assay when used for lung cancer detection on branchial aspirates was 75-81% sensitivity and 90-97% specificity (6, 7), and on FFPE tissue specimens, it had a sensitivity of 89.8% and a specificity of 90.4% (8). Pleural effusion fluid is a very different source of analyte from either branchial lavage or FFPE tissue samples. Pleural effusion is a serious fluid that is seldom mucoid; in most cases, it is sterile, frequently with a hemorrhagic component and usually contains mesothelial cells. According to the recommendations of the user manual for the LungMe^®^ test, 5-20 ml of fresh pleural effusion should be fixed with 20 ml of cell prevention solution to eliminate the interference of mucoid for DNA extraction and the interference of hemoglobin for PCR. The DNA concentration of pleural effusion was sufficient in most cases but was distributed over a very wide range. Through accurate concentration determination, the measurement value of the internal control *ACTB* fluctuated in a very small range, with Ct*_-ACTB_* = 20.48 ± 1.28, which indicated excellent test quality control. In our study, a valid measurement (18 ≤Ct*_-ACTB_* ≤30) was achieved for all 148 specimens, which was obviously improved in comparison to other studies (38.4% to 43.4% invalid results) (16, 17).

Furthermore, the cutoff criterion of the LungMe^®^ methylation assay established for the diagnosis of either branchial lavage or FFPE tissue specimens is not directly transferable to the diagnosis of pleural effusions. Therefore, new cutoff criteria for LungMe^®^ were considered. Samples were classified as methylation positive when at least one of the four genes’ DNA methylation levels correspondingly met the following quantitative criteria: Ct*_SHOX2_* <32 and ΔCt*_SHOX2_* ≤ 9; Ct*_RASSF1A_* <35 and ΔCt*_RASSF1A_* ≤ 12; Ct*_SEPTIN9_* <35 and ΔCt*_SEPTIN9_ ≤*9; or Ct*_HOXA9_* <32 and ΔCt*_HOXA9_* ≤ 8. All others were classified as methylation negative. Of importance, these criteria provide very high specificity (95.8% to 100%) at the expense of sensitivity, especially in the case of *HOXA9*.

Lung cancer is the most common cause of MPE, accounting for approximately 1/3 of MPE cases, followed by BC or OC and GC (3). During the study, we collected 68 MPE cases caused by lung cancer, 14 by BC, 5 by OC, 5 by GC, and 2 by hematonosis, which was consistent with the pathological characteristic of MPE (3). In particular, five retrospective cases of MESO were added to the study. However, the incidence rate of MESO is low in China. Malignant pleural mesothelioma (MPM) is an aggressive cancer of the pleural surface. MPM often presents with recurrent hemorrhagic or inflammatory effusions, which might mask the incipient stages of the disease and thereby delay the diagnosis. The differential diagnosis of benign and malignant mesothelioma is a major challenge for cytopathological investigation (18). Therefore, reliable diagnostic biomarkers are still lacking in MPM.

One of the most frequent underlying causes for MPE is bronchogenic carcinoma, especially peripheral lung adenocarcinoma, close to the pleura (19). Among 68 MPE cases caused by lung cancer, 79.4% (54/64) were diagnosed as adenocarcinoma. Combined with *HOXA9*, the detection rates of LungMe^®^ in adenocarcinoma were further improved from 79.7% to 87.0%. The positive detection rates of *SHOX2* and *RASSF1A* in SCLC reached 100%, which is as good as their performance in BALF and FFPE tissue samples (6–8). Strangely, LungMe^®^ showed a very low detection sensitivity (33%) in MPEs caused by squamous cell carcinoma (LUSC), in contrast to 96.1% sensitivity in FFPE tissue samples. Looking in closer detail at the previous FFPE study, we found that, on the one hand, *SHOX2* primarily contributed to the diagnostic sensitivity in LUSC; on the other hand, the positive rate of *RASSF1A* in LUSC from stage I to stage IV decreased from 76.5% to 22.2%. This could be the reason that the detection rate of RASSF1A was so low (0%) in MPEs caused by metastatic LUSC. In addition, not all cancer patients with pleural effusion appear to have MPE but show paramalignant pleural effusions (PPEs). PPEs can develop in cancer patients due to comorbidities, and they do not contain tumor cells (13, 14). In this study, no tumor cells were found by cytology in 6 LUSC samples, and a low sensitivity was observed for all four methylation markers, which were measured in the cellular fraction of pleural effusion samples. These findings raised the question of whether central LUSC is more likely to cause a PPE. The discrimination between MPE and PPE might direct a decision toward curative or palliative treatment (18). Thus, the highly sensitive methylation analysis might represent a promising ancillary method in addition to cytological analyses in the differential diagnosis between MPE and PPE.

After lung cancer, BC is the second major cause of MPE. Although the four indicators alone have only moderate diagnostic sensitivity (from 35.7% to 57.1) for BC, combinations with OncoMe showed a sensitivity for BC as high as 92.6%. The smallest gene panel (*RASSF1A*, *HOXA9*) showed the same sensitivity of 100% in OC and 80% in MESO compared with the 4-gene panel. Quantitative *SHOX2* and *SEPTIN9* promoter methylation levels have been successfully applied for the detection of malignant cells in pleural effusions (13) and ascites (14). In this study, the combination of *SHOX2* and *SEPTIN9* promoter methylation resulted in an 80% positive diagnostic rate in GC. The limitation of this study is the relatively low number of included patients for some pathological subgroups.

We expect to improve the validation rate of OncoMe with implementation of the assay and increased laboratory experience. In the next step, DNA methylation in both the cellular fraction and supernatant of pleural effusion will be analyzed as an accurate prognostic marker for overall survival in cancer patients with MPE. Furthermore, it is worthwhile to further verify the diagnostic sensitivity of OncoMe in blood samples as pan-cancer markers, especially for lung cancer, BC and OC.

The success rate and detection sensitivity of our assay were obviously improved compared to those in previous studies (13, 16). Our optimized sample preparation and optimal reaction system led to a great increase in sensitivity. DNA methylation-based biomarker tests have been shown to be highly robust and reproducible (8) and therefore can be smoothly implemented into routine clinical practice without the need for highly experienced personnel.

Pleural effusion contained various cell types, including lymphocytes and myeloid cells, etc (20). Genome-scale measures of DNA methylation in samples derived from heterogeneous mixtures of cells, such as pleural effusion, include signals from all cells present (21). Therefore, variation in cell-type proportions across samples has the potential to confound associations of DNA methylation with modeled outcomes. Future research should focus on using new algorithms to infer cellular composition ratios based on DNA methylation data. By using deconvolution approaches to infer underlying cell type proportions, a clearer understanding of independent DNA methylation alterations related to disease, or another outcome. In additional, the human DNA methylation landscape accrues substantial damage over time (22). Various age-related DNA methylation changes have been described, such as differential and variable methylation (23, 24). In this study, we found the diagnostic value of DNA methylation model (LungMe and OncoMe) in young individuals (between 25 and 50 years old) seems higher than in older patients (≥ 50 years old). It also shows the correlation between DNA methylation and age. We still need larger samples to further validate this result.

The difference in sex distribution among different cancer types is one of our limitations, mainly due to our small sample size. Only 100 cancer patients (68 lung cancer, 32 other malignant tumors) were included in this study. We would collect much more clinical samples for further verification in the future. In additional, analyzed samples in OC, GC and MESO are quite low (n=5), which mainly due to the low incidence. Malignant pleural effusion is less common in ovarian and gastric cancer patients. And, MESO is a rare malignancy with few treatment options (25). Next, we aim to collect much more OC, GC and MESO samples.

The lack of NGS-based approaches is one of the important limitations of this study. The DNA methylation levels of SHOX2 and RASSF1A were determined using the NMPA (China National Medical Products Administration) marked *in vitro* diagnostic test. It has been certified in November 2017 by providing clinical data of 1000 cases in three clinical hospitals. In clinical trials, experimental data based on 1000 cases of PCR and NGS comparison data has been provided to prove that the sulfite conversion efficiency is 96.98% (6). The same sulfite conversion kit was used in this experiment. It would suggest that the DNA methylation detection assay based on RT-PCR in this study is credible and shows good consistency with NGS-based approaches.

As well-known, most malignant pleural effusions are secondary to metastases to the pleura, most often from lung or breast cancer. Along with the identification of MPE, identifying the source of the metastases to the pleura is critical to the treatment and prognosis of the patient. Our panel (OncoMe) cannot identify specific cancer type is the important limitation of this study. In future studies, we will further explore more tumor-specific methylation markers to establish new panels to identify specific cancer type.

In conclusion, we found that the aberrant promotor methylation of OncoMe (*SHOX2*, *RASSF1A*, *SEPTIN9* and *HOXA9*) is a cancer-specific alteration and may be a valuable marker to aid in the differentiation of MPE, even not limited to lung cancer. OncoMe routine testing could help clinicians quickly identify MPE and facilitate clinical decision-making.

## Data availability statement

The original contributions presented in the study are included in the article/**Supplementary Material**. Further inquiries can be directed to the corresponding author.

## Ethics statement

The studies involving human participants were reviewed and approved by the First Hospital of China Medical University (Reference number: AF-SOP-07-1.1-01). Written informed consent for participation was not required for this study in accordance with the national legislation and the institutional requirements.

## Author contributions

Conceptualization: GH; data curation: CL, NL, and QZ; methodology: MD, JM, and JL; project administration: YY, JW, YM, and QL; writing - original draft: BS and GH; writing - review and editing: BS and GH. All authors contributed to the article and approved the submitted version.

## Funding

This research was supported by National High Level Hospital Clinical Research Funding(2022-NHLHCRF-LX-01), the Non-profit Central Research Institute Fund of Chinese Academy of Medical Sciences (No. 2020-PT320-001), Elite Medical Professionals project of China-Japan Friendship Hospital (NO. ZRJY2021- BJ08), Shanghai Pudong New District Foundation for Development of Science and Technology (PKX2021-S09).

## Conflict of interest

BS was employed by the company Tellgen Corporation, Shanghai, China.

The remaining authors declare that the research was conducted in the absence of any commercial or financial relationships that could be construed as a potential conflict of interest.

## Publisher’s note

All claims expressed in this article are solely those of the authors and do not necessarily represent those of their affiliated organizations, or those of the publisher, the editors and the reviewers. Any product that may be evaluated in this article, or claim that may be made by its manufacturer, is not guaranteed or endorsed by the publisher.

## Supplementary material

Supplementary Figure 1 Frequency distribution of pleural fluid concentration.

Supplementary Figure 2 Amplification curve of *HOXA9, RASSF1A, SHOX2*, and *SEPTIN9* methylation. **(A)** Amplification curve of HOXA9 was evaluated by analyzing 6 different relative HOXA9 concentration (0.4%, 0.8%, 1.6%, 6.4%, 25%,100%) with a total DNA concentration indicated by CtIC(20:2500copies/ul); **(B)** Amplification curve of RASSF1A was evaluated by analyzing 6 different relative RASSF1A concentration (0.4%, 0.8%, 1.6%, 6.4%, 25%,100%) with a total DNA concentration indicated by CtIC(20:2500copies/ul); **(C)** Amplification curve of SHOX2 was evaluated by analyzing 6 different relative SHOX2 concentration (0.4%, 0.8%, 1.6%, 6.4%, 25%,100%) with a total DNA concentration indicated by CtIC(20:2500copies/ul); **(D)** Amplification curve of SEPTIN9 was evaluated by analyzing 6 different relative SEPTIN9 concentration (0.4%, 0.8%, 1.6%, 6.4%, 25%,100%) with a total DNA concentration indicated by CtIC(20:2500copies/ul); five PCR replicates pre sample were performed.

Supplementary Figure 3 ROC curves for the LungMe methylation panels in older patients (≥ 50 years old) **(A)**, and in young individuals (between 25 and 50 years old) **(B).** ROC curves for the OncoMe methylation panels in older patients (≥ 50 years old) **(C)**, and in young individuals (between 25 and 50 years old) **(D).**

## Abbreviations

MPE, malignant pleural effusion; SHOX2, short stature homeobox gene two; RASSF1A, RAS association domain family 1, isoform A; HOXA9, homeobox A9; SEPTIN9, Septin 9; LUAC, lung adenocarcinoma; LUSC, lung squamous carcinoma; SCLC, small cell lung carcinoma; BC, breast cancer; OC, ovarian carcinoma; GC, gastrointestinal cancer; MESO, malignant mesothelioma; PNE, pneumonia; TB, tuberculous pleuritis; MS-PCR, methylation-specific real-time PCR; ROC, receiver operating characteristic; AUC, the area under the ROC curve; BPE, benign pleural effusion.
