# Supplementary figures and images for: A detection panel of novel methylated DNA markers for malignant pleural effusion
Source: Front Oncol. 2022 Sep 13;12:967079. doi: 10.3389/fonc.2022.967079 (PMC9513209; doi:10.3389/fonc.2022.967079)

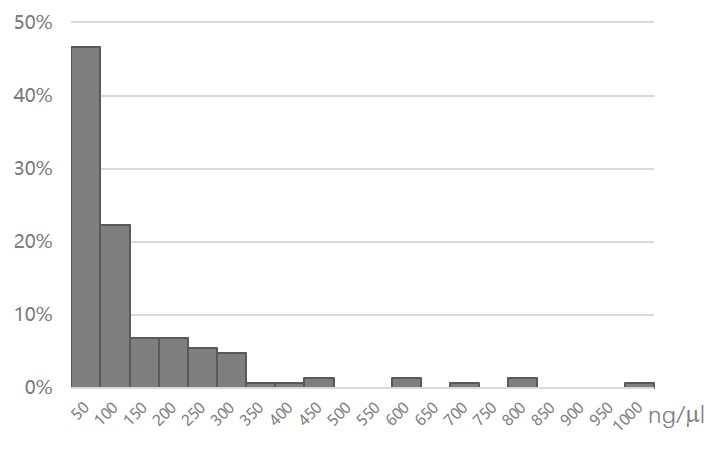

Supplement: Supplementary Figure 1 — Frequency distribution of pleural fluid concentration. [file Image_1.jpeg]

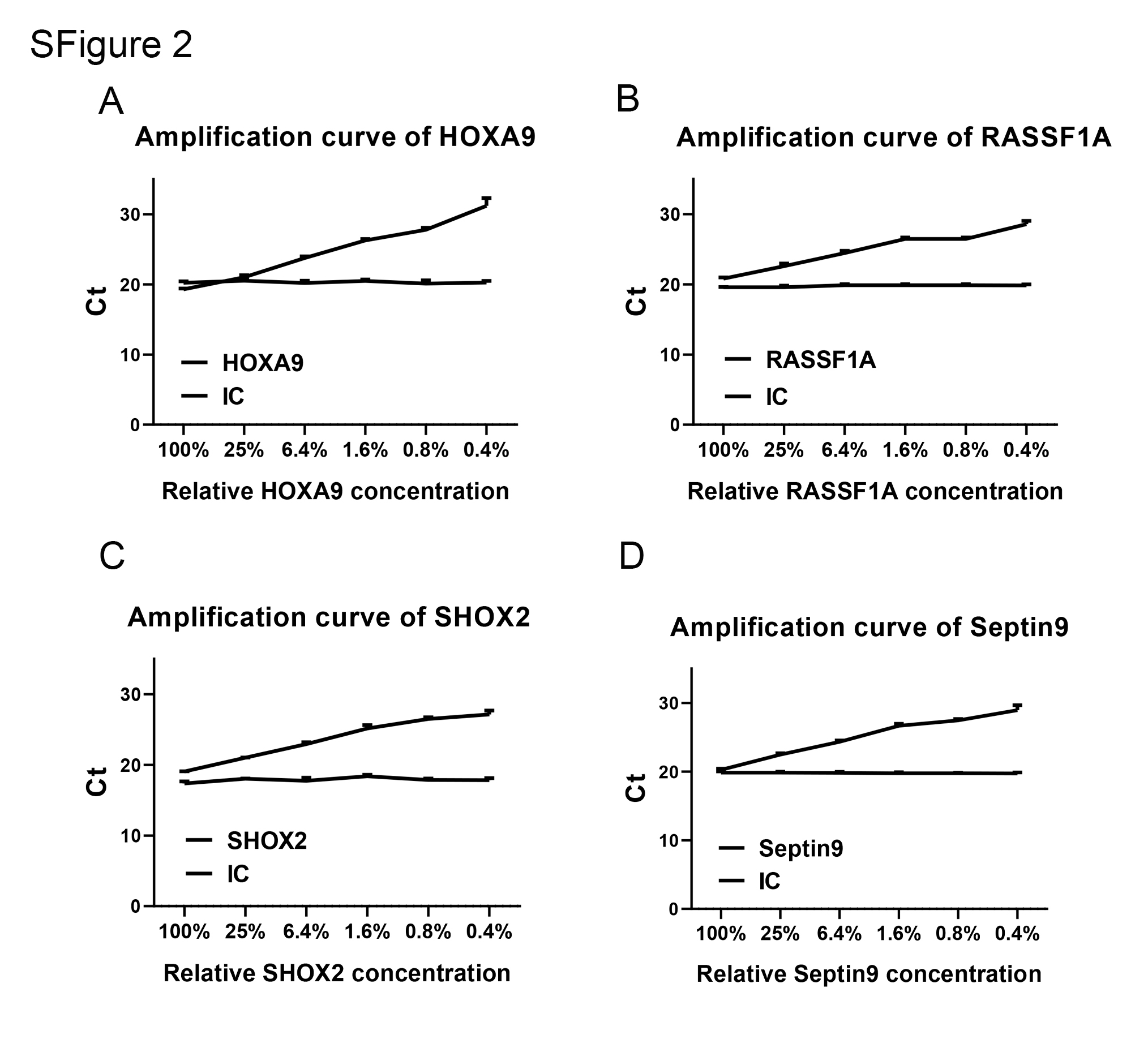

Supplement: Supplementary Figure 2 — Amplification curve of HOXA9, RASSF1A, SHOX2, and SEPTIN9 methylation. (A) Amplification curve of HOXA9 was evaluated by analyzing 6 different relative HOXA9 concentration (0.4%, 0.8%, 1.6%, 6.4%, 25%,100%) with a total DNA concentration indicated by CtIC(20:2500copies/ul); (B) Amplification curve of RASSF1A was evaluated by analyzing 6 different relative RASSF1A concentration (0.4%, 0.8%, 1.6%, 6.4%, 25%,100%) with a total DNA concentration indicated by CtIC(20:2500copies/ul); (C) Amplification curve of SHOX2 was evaluated by analyzing 6 different relative SHOX2 concentration (0.4%, 0.8%, 1.6%, 6.4%, 25%,100%) with a total DNA concentration indicated by CtIC(20:2500copies/ul); (D) Amplification curve of SEPTIN9 was evaluated by analyzing 6 different relative SEPTIN9 concentration (0.4%, 0.8%, 1.6%, 6.4%, 25%,100%) with a total DNA concentration indicated by CtIC(20:2500copies/ul); five PCR replicates pre sample were performed. [file Image_2.jpeg]

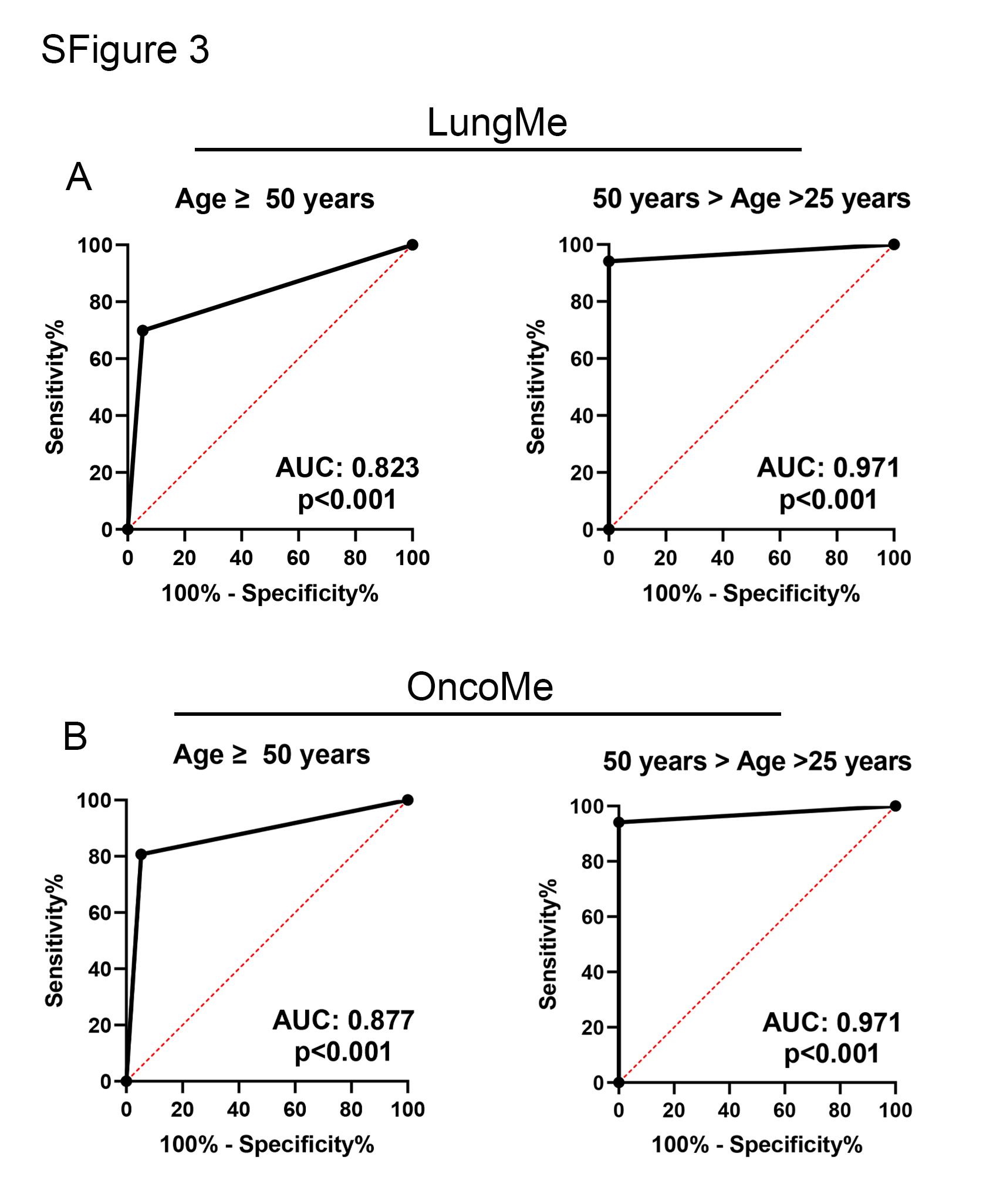

Supplement: Supplementary Figure 3 — ROC curves for the LungMe methylation panels in older patients (≥ 50 years old) (A), and in young individuals (between 25 and 50 years old) (B). ROC curves for the OncoMe methylation panels in older patients (≥ 50 years old) (C), and in young individuals (between 25 and 50 years old) (D). [file Image_3.jpeg]
